# Supplementary material for: Integrative analysis of transcriptome dynamics during human craniofacial development identifies candidate disease genes
Source: Nat Commun. 2023 Aug 2;14:4623. doi: 10.1038/s41467-023-40363-1 (PMC10397224; doi:10.1038/s41467-023-40363-1)
Supplement: Supplementary file 1 — Supplementary Information [file 41467_2023_40363_MOESM1_ESM.pdf]

Figure S1

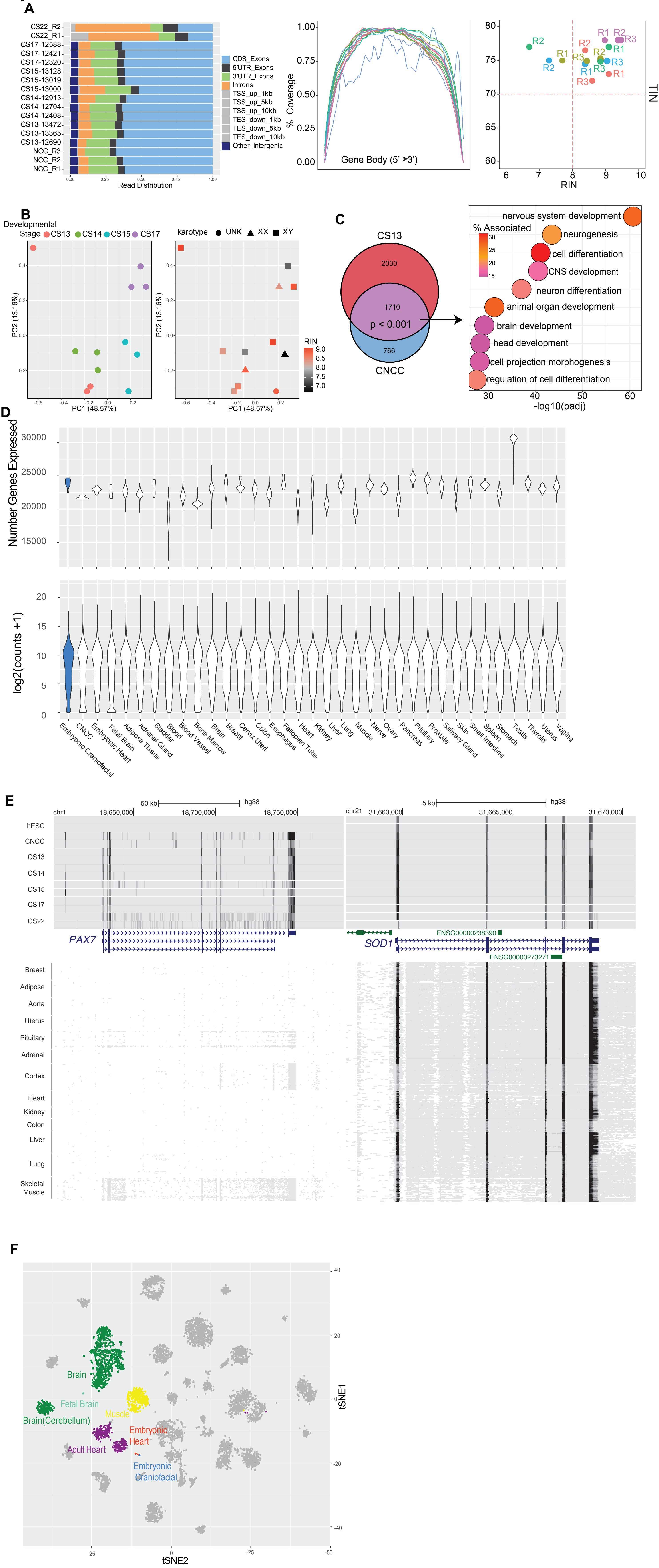
A
Read Distribution
CDS_Exons
5'UTR_Exons
3'UTR_Exons
Introns
TSS_up_1kb
TSS_up_5kb
TSS_up_10kb
TES_down_1kb
TES_down_5kb
TES_down_10kb
Other_intergenic
% Coverage
Gene Body (5' → 3')
RIN
TIN
B
Developmental Stage
CS13
CS14
CS15
CS17
karotype
UNK
XX
XY
PC1 (48.57%)
PC2 (13.16%)
RIN
C
CS13
CNCC
2030
1710
766
p < 0.001
% Associated
nervous system development
neurogenesis
cell differentiation
CNS development
neuron differentiation
animal organ development
brain development
head development
cell projection morphogenesis
regulation of cell differentiation
-log10(padj)
D
Number Genes Expressed
log2(counts +1)
Embryonic Craniofacial
CNCC
Embryonic Heart
Fetal Brain
Adipose Tissue
Adrenal Gland
Bladder
Blood
Blood Vessel
Bone Marrow
Brain
Breast
Cervix Uteri
Colon
Esophagus
Fallopian Tube
Heart
Kidney
Liver
Lung
Muscle
Nerve
Ovary
Pancreas
Pituitary
Prostate
Salivary Gland
Skin
Small Intestine
Spleen
Stomach
Testis
Thyroid
Uterus
Vagina
E
PAX7
SOD1
hESC
CNCC
CS13
CS14
CS15
CS17
CS22
Breast
Adipose
Aorta
Uterus
Pituitary
Adrenal
Cortex
Heart
Kidney
Colon
Liver
Lung
Skeletal Muscle
F
Brain
Fetal Brain
Brain(Cerebellum)
Muscle
Embryonic Heart
Adult Heart
Embryonic Craniofacial
tSNE1
tSNE2

Figure S1. Developing craniofacial tissue quality control metrics and global multi-tissue comparison. A. Quality control metrics from left to right. Read distribution plot generated by RSeQC. PolyA RNA samples dominated by CDS_exons while the two total-RNA samples (CS22) dominated by Intronic regions as expected. Gene body coverage plot show reads lack a 3' bias. RIN vs. TIN plot. TIN values established by (Wang et al., 2016) were calculated using RSeQC and are all above the recommended threshold of 70. **B.** PCA plots of global gene expression for bulk RNA primary tissue samples generated in this study. The left indicates CS staging. The right plot marks karyotyping and RIN values for each sample, XX (female), XY (male), UNK (unknown). **C.** Venn diagram of upregulated genes in CNCCs or CS13 compared to H9 ESCs (p-adj <0.05, log2FoldChange >0.5). The same number of genes were randomly chosen from ~10,000 upregulated genes (padj < 0.05, log2FoldChange >0) 1000x to get a permutation p-value of the number of expected intersecting genes. The dot plot is the gene ontology of some of the top most significant terms using GO David. The color bar is the % of genes from the input list that are associated with that GO term. **D.** Comparison of raw counts from GTEx with early developing tissues and CNCCs. The top plot is the number of genes above 10 counts per sample per tissue. The bottom plot is the gene expression ranges for the average expression across all samples per tissue. **E.** Genome browser shot of *PAX7* and *SOD1* bulk RNA-seq signal across several tissues. *PAX7* expression is heavily biased in the craniofacial tissue and CNCCs. **F.** tSNE plot of global gene expression counts for multi-tissue analysis (craniofacial, embryonic heart, fetal brain, GTEx). Representative tissues are highlighted by color: craniofacial (blue), embryonic heart (red), fetal brain (light green), GTEx brain (green), GTEx Heart (purple), GTEx muscle (yellow), all other GTEx tissues (grey).

Figure S2

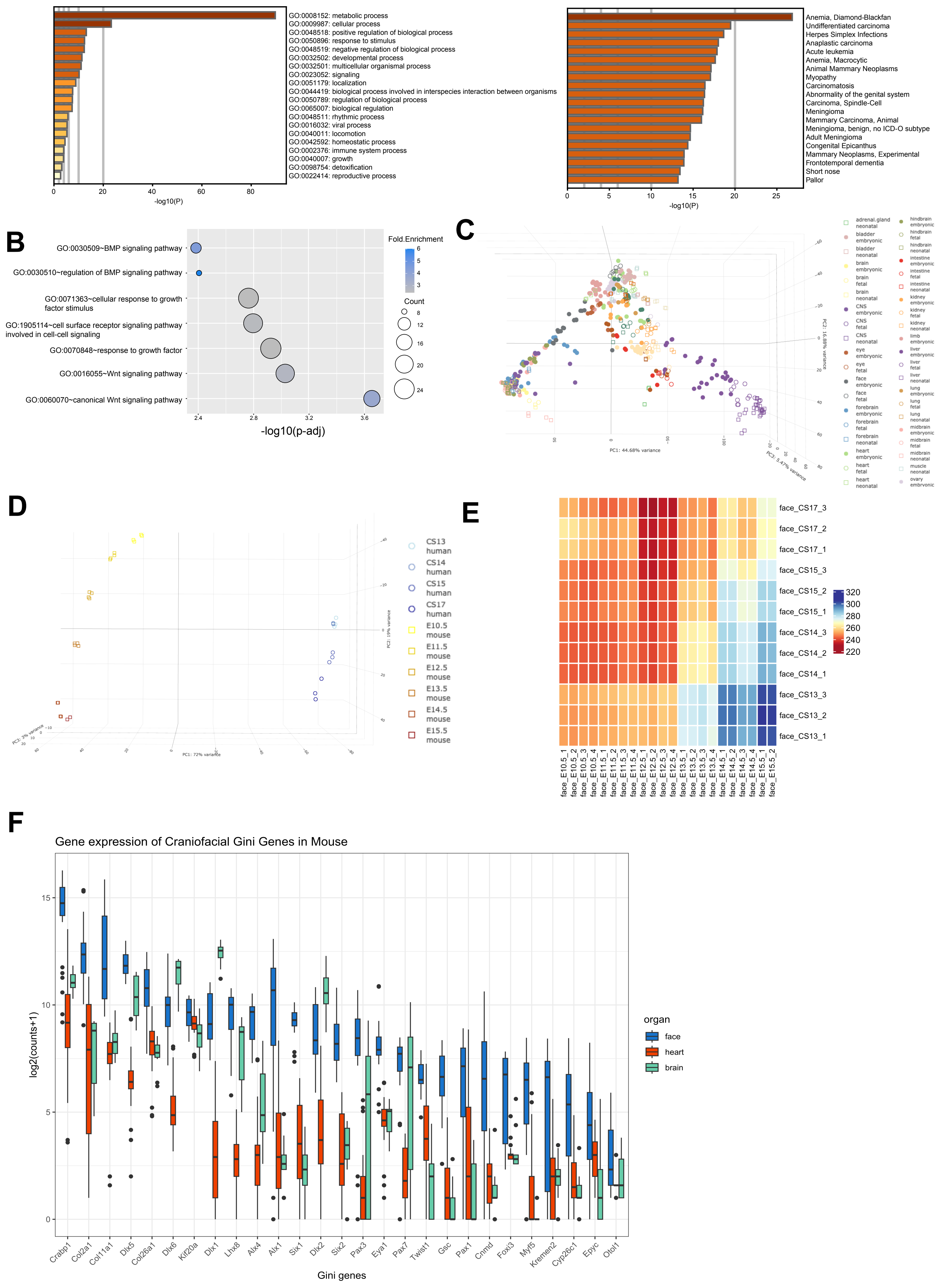

A
GO Enrichment
GO:0008152: metabolic process
GO:0009987: cellular process
GO:0048518: positive regulation of biological process
GO:0050896: response to stimulus
GO:0048519: negative regulation of biological process
GO:0032502: developmental process
GO:0032501: multicellular organismal process
GO:0023052: signaling
GO:0051179: localization
GO:0044419: biological process involved in interspecies interaction between organisms
GO:0050789: regulation of biological process
GO:0065007: biological regulation
GO:0048511: rhythmic process
GO:0016032: viral process
GO:0040011: locomotion
GO:0042592: homeostatic process
GO:0002376: immune system process
GO:0040007: growth
GO:0098754: detoxification
GO:0022414: reproductive process
-log10(P)
Disease Enrichment
Anemia, Diamond-Blackfan
Undifferentiated carcinoma
Herpes Simplex Infections
Anaplastic carcinoma
Acute leukemia
Anemia, Macrocytic
Animal Mammary Neoplasms
Myopathy
Carcinomatosis
Abnormality of the genital system
Carcinoma, Spindle-Cell
Meningioma
Mammary Carcinoma, Animal
Meningioma, benign, no ICD-O subtype
Adult Meningioma
Congenital Epicanthus
Mammary Neoplasms, Experimental
Frontotemporal dementia
Short nose
Pallor
-log10(P)
B
GO:0030509~BMP signaling pathway
GO:0030510~regulation of BMP signaling pathway
GO:0071363~cellular response to growth factor stimulus
GO:1905114~cell surface receptor signaling pathway involved in cell-cell signaling
GO:0070848~response to growth factor
GO:0016055~Wnt signaling pathway
GO:0060070~canonical Wnt signaling pathway
Fold.Enrichment
Count
-log10(p-adj)
C
PC1: 44.68% variance
PC2: 16.88% variance
PC3: 5.47% variance
D
CS13 human
CS14 human
CS15 human
CS17 human
E10.5 mouse
E11.5 mouse
E12.5 mouse
E13.5 mouse
E14.5 mouse
E15.5 mouse
PC1: 72% variance
PC2: 19% variance
PC3: 3% variance
E
face_CS17_3
face_CS17_2
face_CS17_1
face_CS15_3
face_CS15_2
face_CS15_1
face_CS14_3
face_CS14_2
face_CS14_1
face_CS13_3
face_CS13_2
face_CS13_1
F
Gene expression of Craniofacial Gini Genes in Mouse
log2(counts+1)
Gini genes
organ
face
heart
brain
Crabp1
Col2a1
Col11a1
Dlx5
Col26a1
Dlx6
Kif20a
Dlx1
Lhx8
Alx4
Alx1
Six1
Dlx2
Six2
Pax3
Eya1
Pax7
Twist1
Gsc
Pax1
Cnmd
Foxl3
Myf5
Kremen2
Cyp26c1
Epyc
Otol1

Figure S2. Signaling-related gene ontologies of craniofacial Gini genes.
A. Gene ontology and disease enrichments for 239 genes with highest absolute expression in craniofacial tissues without regard to other tissues. **B.** Selected set of significant (Benjamini and Hochberg–adjusted p-value < 0.05) gene ontologies for Gini genes that involved signaling. The dot size or count indicates the number of genes within a disease category. Color bars are log2 of the fold enrichment (see Methods). C. three-dimensional (3D) plot of Principal Component Analysis (PCA) from bulk RNA-sequencing data of 500 mouse embryonic samples. Three major components are used for plot. Each color represents tissue types with open shapes indicating fetal and postnatal tissues and filled shapes indicating embryonic tissues. Developmental periods are roughly distinguished by 3 groups: embryonic (E10.5~E16); fetal (E16~E21); and neonatal (after P00). D. three-dimensional (3D) plot of Principal Component Analysis (PCA) from bulk RNA-sequencing data of human embryonic craniofacial samples (n=12) and mouse embryonic facial prominence samples (n=22). 3 major components are used for plot. E. Euclidean distances from pairwise-comparisons of gene expression of one-to-one orthologous genes (n=15214) for human and mice facial samples.F. Boxplots of gene expression from indicated mouse tissues for genes with craniofacial gini status in both human and mouse. Face (n=22) Heart (n=36) Brain (n=116) .The centre line denotes the median value (50th percentile), the box contains the 25th to 75th percentiles and the whiskers mark the 5th and 95th percentiles. Data points beyond these values (outliers) are shown as black dots.

Figure S3

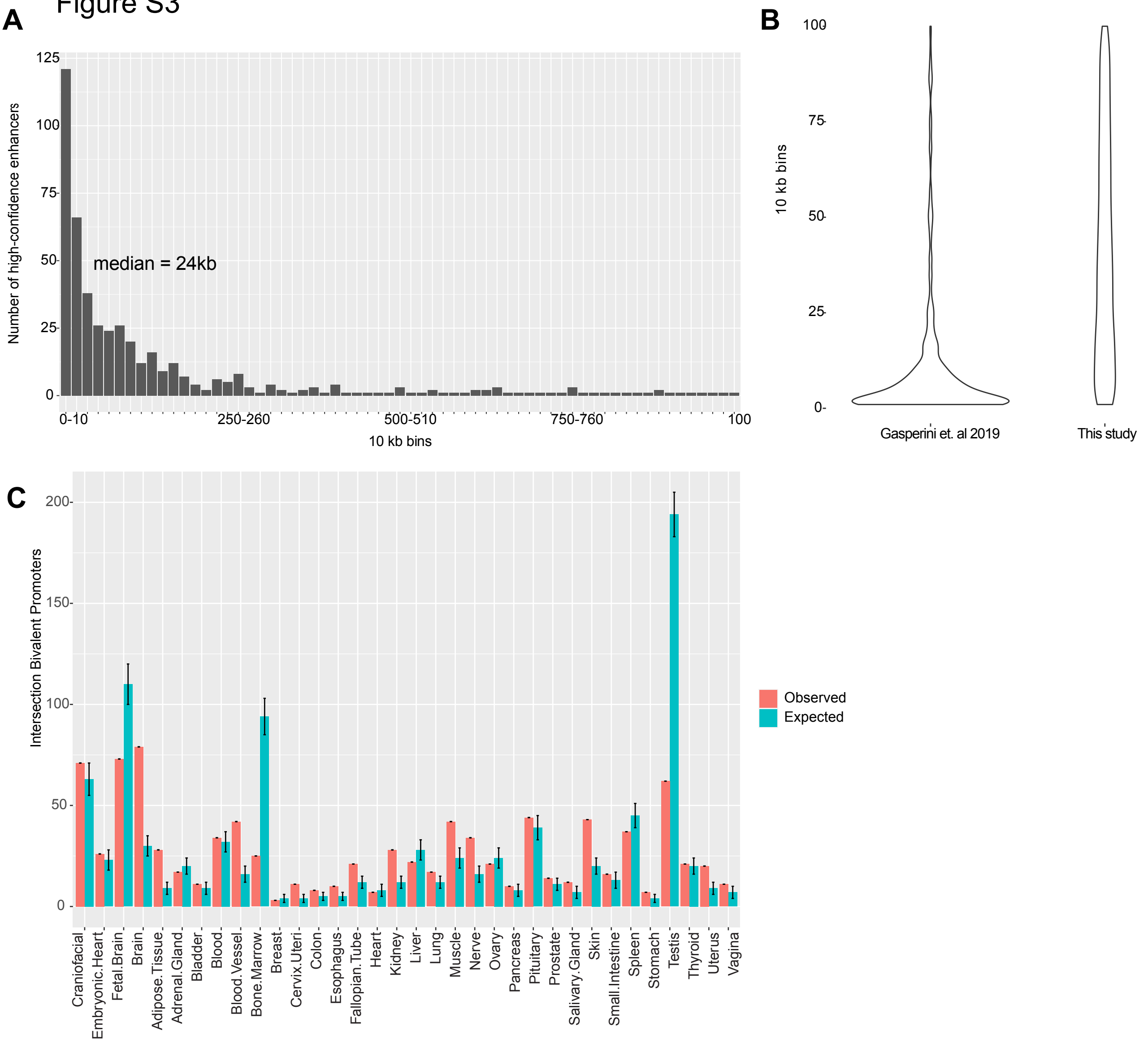

A
Number of high-confidence enhancers
median = 24kb
10 kb bins
0-10
250-260
500-510
750-760
100
B
10 kb bins
Gasperini et. al 2019
This study
C
Intersection Bivalent Promoters
Observed
Expected
Craniofacial
Embryonic.Heart
Fetal.Brain
Brain
Adipose.Tissue
Adrenal.Gland
Bladder
Blood
Blood.Vessel
Bone.Marrow
Breast
Cervix.Uteri
Colon
Esophagus
Fallopian.Tube
Heart
Kidney
Liver
Lung
Muscle
Nerve
Ovary
Pancreas
Pituitary
Prostate
Salivary.Gland
Skin
Small.Intestine
Spleen
Stomach
Testis
Thyroid
Uterus
Vagina

Figure S3. Gene-enhancer pair distances are larger for developing tissue. A. Histogram plot of distances for high-confidence gene-enhancer pairs for K562 cells from Gasperini et al. 2019[150]. **B.** Violin plots comparing the distances for our CF Gini gene-CFSE pairs to Gasperini data[150] (see Methods). **C.** Overlap of gini genes across all tissues for genes with bivalent promoters in craniofacial tissue. The bars for the expected overlap represent the median and lines represent standard deviation calculated from 1000 iterations of randomly selected gini genes (n=5134).

Figure S4

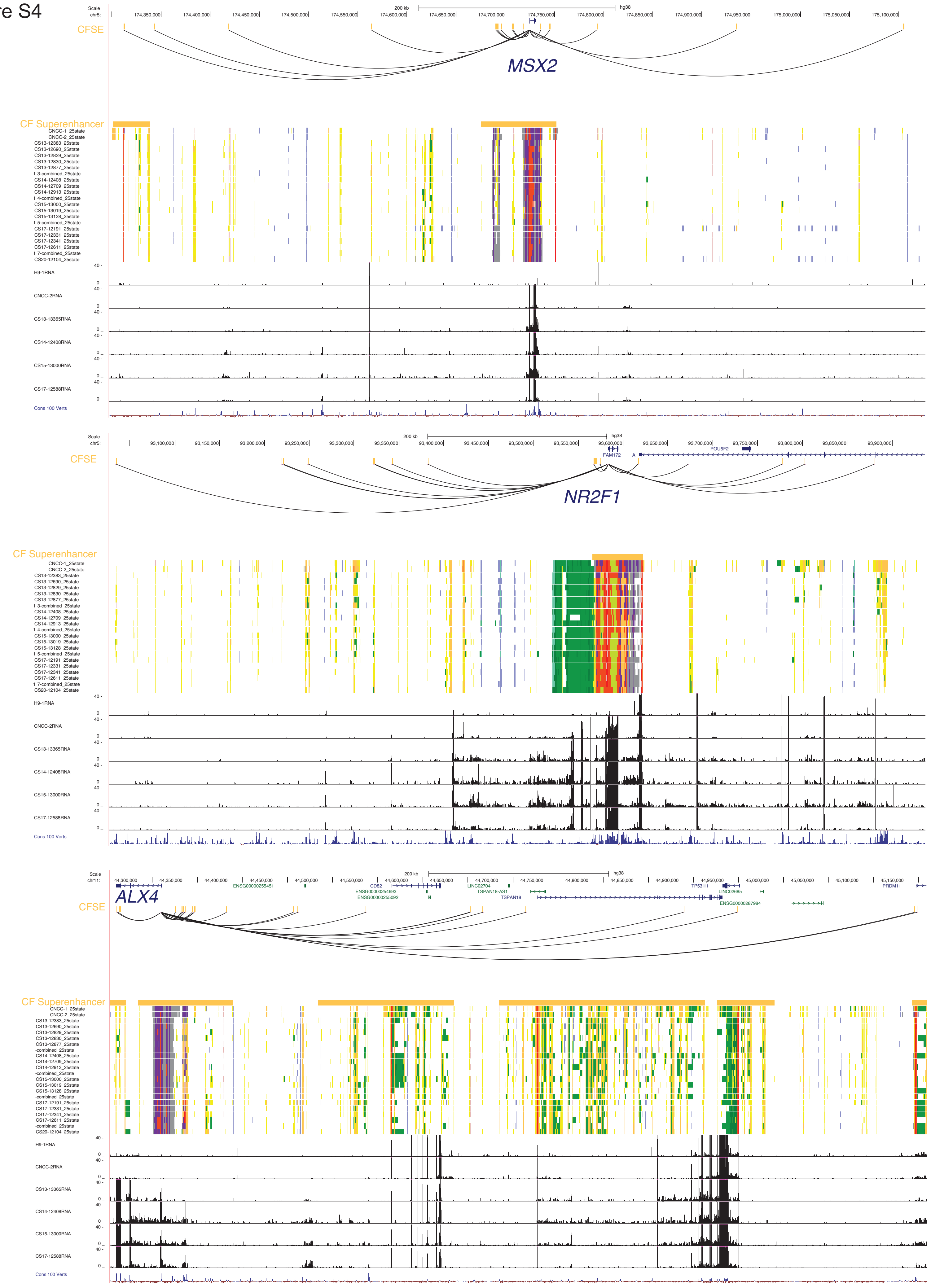
MSX2
NR2F1
ALX4
CFSE
CF Superenhancer
H9-1RNA
CNCC-2RNA
CS13-13365RNA
CS14-12408RNA
CS15-13000RNA
CS17-12588RNA
Cons 100 Verts

Figure S4. Developmental craniofacial transcription factors genome browser shots. Genome browser shot of gene locus showing 25 state ChromHMM segmentation from culture model of CNCCs, and CS13 through CS20 of primary human craniofacial tissue. The dark purple indicates bivalent state which are located near or at the promoter (reds). Above the segmentations track is the active and CFSE track shown in orange which include strong enhancer ChromHMM states 13-15. Below are RNA-seq bigwig signal tracks for human embryonic stem cells (ESC), CNCCs, and CS13 through CS22. *Top* NR2F1 *middle* MSX2 *bottom* ALX4.

Figure S5

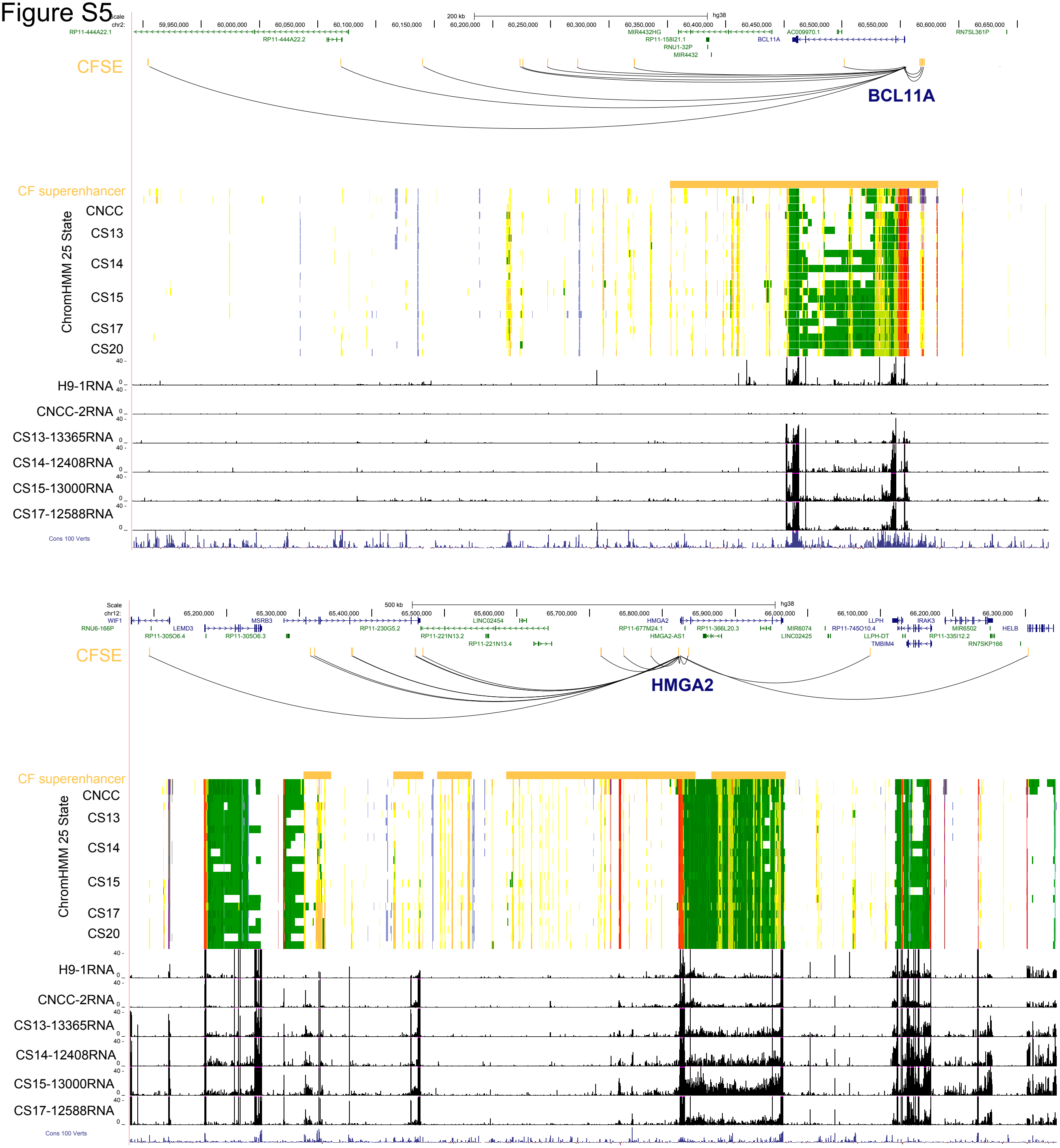

Scale
chr2:
200 kb
hg38
59,950,000
60,000,000
60,050,000
60,100,000
60,150,000
60,200,000
60,250,000
60,300,000
60,350,000
60,400,000
60,450,000
60,500,000
60,550,000
60,600,000
60,650,000
RP11-444A22.1
RP11-444A22.2
MIR4432HG
RP11-158I21.1
RNU1-32P
MIR4432
BCL11A
AC009970.1
RN7SL361P
CFSE
BCL11A
CF superenhancer
ChromHMM 25 State
CNCC
CS13
CS14
CS15
CS17
CS20
H9-1RNA
CNCC-2RNA
CS13-13365RNA
CS14-12408RNA
CS15-13000RNA
CS17-12588RNA
40 -
0 _
Cons 100 Verts
Scale
chr12:
500 kb
hg38
65,200,000
65,300,000
65,400,000
65,500,000
65,600,000
65,700,000
65,800,000
65,900,000
66,000,000
66,100,000
66,200,000
66,300,000
WIF1
RNU6-166P
LEMD3
RP11-305O6.4
MSRB3
RP11-305O6.3
RP11-230G5.2
LINC02454
RP11-221N13.2
RP11-221N13.4
HMGA2
RP11-677M24.1
HMGA2-AS1
RP11-366L20.3
MIR6074
LINC02425
LLPH
RP11-745O10.4
LLPH-DT
TMBIM4
IRAK3
MIR6502
RP11-335I12.2
RN7SKP166
HELB
CFSE
HMGA2
CF superenhancer
ChromHMM 25 State
CNCC
CS13
CS14
CS15
CS17
CS20
H9-1RNA
CNCC-2RNA
CS13-13365RNA
CS14-12408RNA
CS15-13000RNA
CS17-12588RNA
40 -
0 _
Cons 100 Verts

Figure S5. Putative developmental craniofacial transcription factors genome browser shots. Genome browser shot of gene locus showing 25 state ChromHMM segmentation from culture model of CNCCs, and CS13 through CS20 of primary human craniofacial tissue. The dark purple indicates bivalent state which are located near or at the promoter (reds). Above the segmentations track is the active and novel CFSE track shown in orange which include strong enhancer ChromHMM states 13-15. Below are RNA-seq bigwig signal tracks for human embryonic stem cells (ESC), CNCCs, and CS13 through CS22. *Top* BCL11A *bottom* HMGA2.

Figure S6

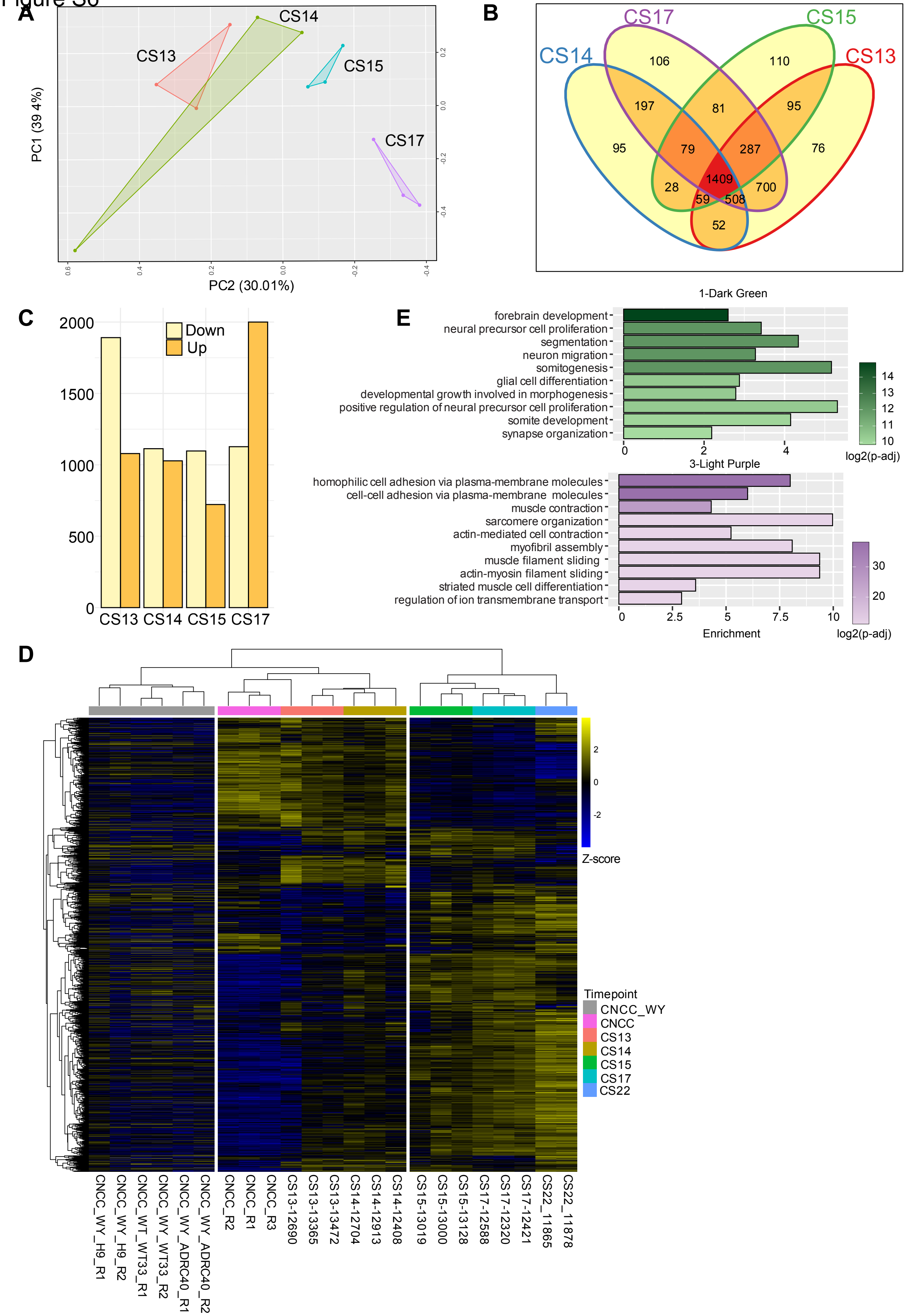
A
CS14
CS13
CS15
CS17
PC1 (39.4%)
PC2 (30.01%)
B
CS17
CS15
CS14
CS13
106
110
197
81
95
95
79
287
76
1409
28
700
59
508
52
C
2000
1500
1000
500
0
Down
Up
CS13 CS14 CS15 CS17
E
1-Dark Green
forebrain development
neural precursor cell proliferation
segmentation
neuron migration
somitogenesis
glial cell differentiation
developmental growth involved in morphogenesis
positive regulation of neural precursor cell proliferation
somite development
synapse organization
log2(p-adj)
3-Light Purple
homophilic cell adhesion via plasma-membrane molecules
cell-cell adhesion via plasma-membrane molecules
muscle contraction
sarcomere organization
actin-mediated cell contraction
myofibril assembly
muscle filament sliding
actin-myosin filament sliding
striated muscle cell differentiation
regulation of ion transmembrane transport
Enrichment
log2(p-adj)
D
Z-score
Timepoint
CNCC_WY
CNCC
CS13
CS14
CS15
CS17
CS22
CNCC_WY_H9_R1
CNCC_WY_H9_R2
CNCC_WT_WT33_R1
CNCC_WY_WT33_R2
CNCC_WY_ADRC40_R1
CNCC_WY_ADRC40_R2
CNCC_R2
CNCC_R1
CNCC_R3
CS13-12690
CS13-13365
CS13-13472
CS14-12704
CS14-12913
CS14-12408
CS15-13019
CS15-13000
CS15-13128
CS17-12588
CS17-12320
CS17-12421
CS22_11865
CS22_11878

Figure S6. Differential expression of early developing craniofacial tissues.
A. PCA plot of RNA-seq samples generated in this study including only differentially expressed genes. **B.** Venn diagram of differentially expressed genes**. C.** Bar plots showing number of up and down regulated differentially expressed genes compared to each time point. For example CS17 has 2000 up regulated and 1000 down regulated genes in pairwise comparisons with CS13, CS14 and CS15. **D.** Heatmap of differentially expressed genes (rows) separated into three panels. The first panel showcases the data from Prescott et al., 2015[42]**.** The other panels are separating samples into early (CNCC, CS13,CS14) and late (CS15, CS17, CS22). Related to Figure 4A. **E.** Gene ontology of a select subset of the most significant terms identified by clusterProfiler[147] for 1-Dark Green and 3-Light Purple. The genes belonging to 1-Dark Green are enriched for functions relating to neurogenesis and somitogenesis. The light purple has a focus on cell-cell adhesion and sarcomere organization. Related to Figure 4B.

Figure S7

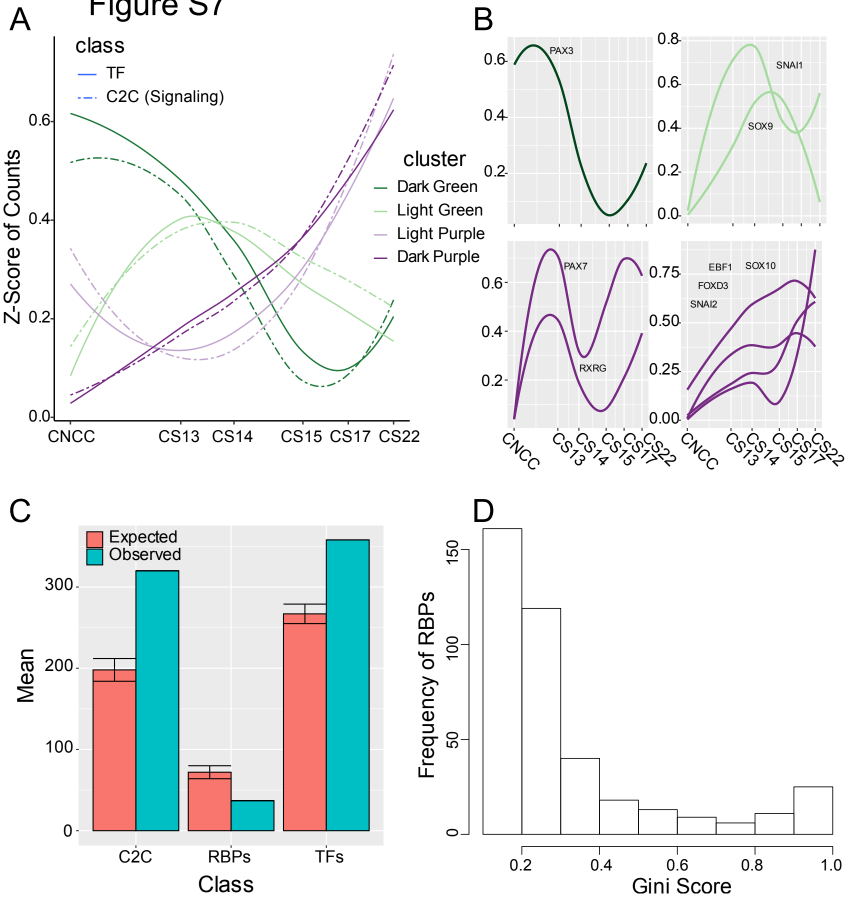
A
class
TF
C2C (Signaling)
cluster
Dark Green
Light Green
Light Purple
Dark Purple
Z-Score of Counts
0.0
0.2
0.4
0.6
CNCC
CS13
CS14
CS15
CS17
CS22
B
PAX3
SNAI1
SOX9
PAX7
RXRG
EBF1
SOX10
FOXD3
SNAI2
C
Expected
Observed
Mean
0
100
200
300
C2C
RBPs
TFs
Class
D
Frequency of RBPs
0
50
100
150
0.2
0.4
0.6
0.8
1.0
Gini Score

Figure S7. Gene class analysis of differentially expressed genes. A. Trajectories of the z-score of the raw counts of all differentially expressed genes separated by class, TF or C2C (cell-cell signaling) and dendrogram cluster from Figure 4A. **B.** Trajectories of the z-score of the raw counts of single genes colored by the dendrogram cluster from Figure 4A. Genes in Dark Purple-4 have two separate plots to highlight differences. **C.** Bar plots of expected versus observed number of genes from each gene class. The bars for the expected overlap represent the mean and lines represent standard deviation calculated from 1000 iterations of randomly selected genes from the background set (n=18597). **D.** Histogram of Gini scores of all RBPs (n=415).

Figure S8

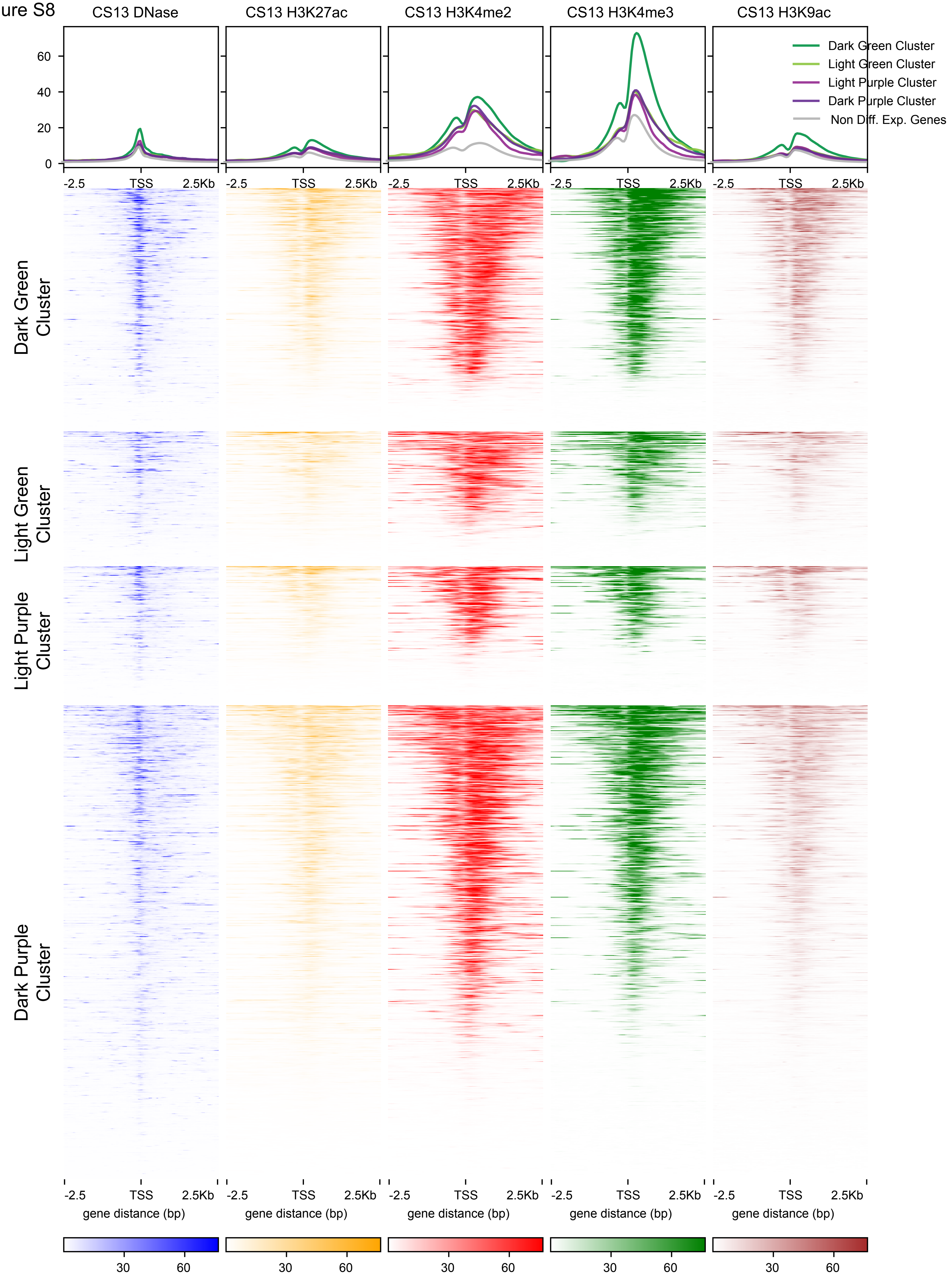

CS13 DNase
CS13 H3K27ac
CS13 H3K4me2
CS13 H3K4me3
CS13 H3K9ac
Dark Green Cluster
Light Green Cluster
Light Purple Cluster
Dark Purple Cluster
Non Diff. Exp. Genes
0
20
40
60
-2.5
TSS
2.5Kb
Dark Green Cluster
Light Green Cluster
Light Purple Cluster
Dark Purple Cluster
gene distance (bp)
30
60

Figure S8. ChIP-seq signal across differentially expressed genes from multiple histone modifications. The ChIP data is from CS13 human primary tissue. Note that genes of the Dark Green-1 cluster have the highest signal from H3K4me3, an epigenetic marker for activation of transcription. These genes were also shown to have the highest expression in our early tissues (Figure 4A).

Figure S9

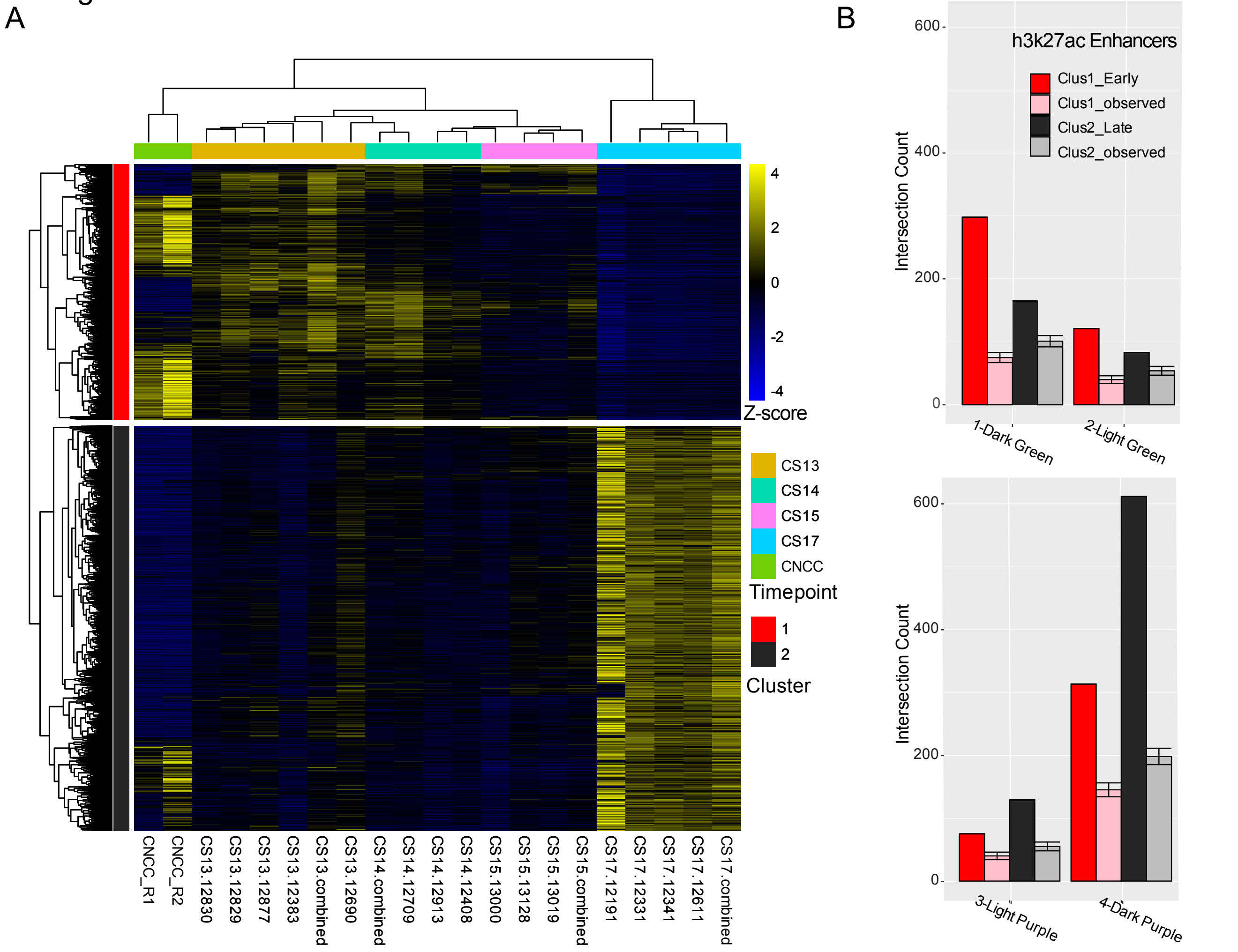
A
B
Z-score
4
2
0
-2
-4
CS13
CS14
CS15
CS17
CNCC
Timepoint
1
2
Cluster
CNCC_R1
CNCC_R2
CS13.12830
CS13.12829
CS13.12877
CS13.12383
CS13.combined
CS13.12690
CS14.combined
CS14.12709
CS14.12913
CS14.12408
CS15.13000
CS15.13128
CS15.13019
CS15.combined
CS17.12191
CS17.12331
CS17.12341
CS17.12611
CS17.combined
h3k27ac Enhancers
Clus1_Early
Clus1_observed
Clus2_Late
Clus2_observed
Intersection Count
600
400
200
0
1-Dark Green
2-Light Green
3-Light Purple
4-Dark Purple

Figure S9. Differential H3K27ac signal between CS13 and CS17. A. Heatmap of H3K27ac segments (n=19010) separated into two clusters through hierarchical clustering. The Z-score indicates signal intensity where yellow is high and blue is low. Cluster 1 tends to have higher signal in the early developmental stages and Cluster 2 has higher signal in the later developmental stage (CS17). **B.** The intersection of differentially expressed genes from the clusters in Figure 4A and genes obtained by assigning H3K27ac segments using GREAT. Cluster 1 is more enriched for genes upregulated in the early stages (Dark Green-1) and cluster 2 for late stages (Dark Purple-4). The light pink and light gray bars represent the median of the expected number of overlaps and lines are standard deviations calculated from 1000 iterations of randomly selected genes from the background set (n=18597).

Figure S10

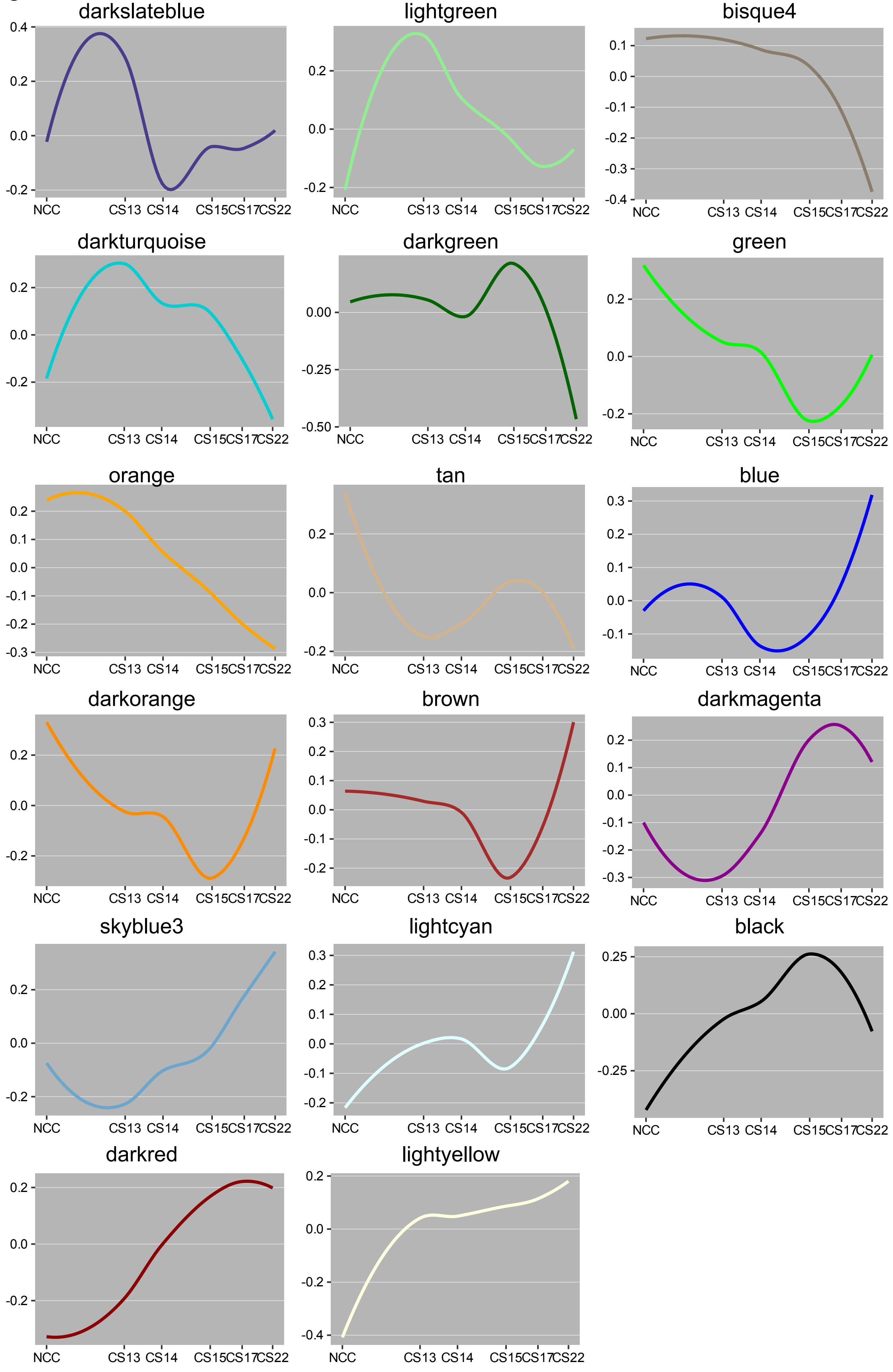
darkslateblue
lightgreen
bisque4
darkturquoise
darkgreen
green
orange
tan
blue
darkorange
brown
darkmagenta
skyblue3
lightcyan
black
darkred
lightyellow
NCC
CS13
CS14
CS15
CS17
CS22

Figure S10. Trajectory plots for WGCNA module eigengenes. Related to Figure 5B. Trajectories of expression based on eigengene vectors reported by WGCNA for each module across the developmental time series.

Figure S11

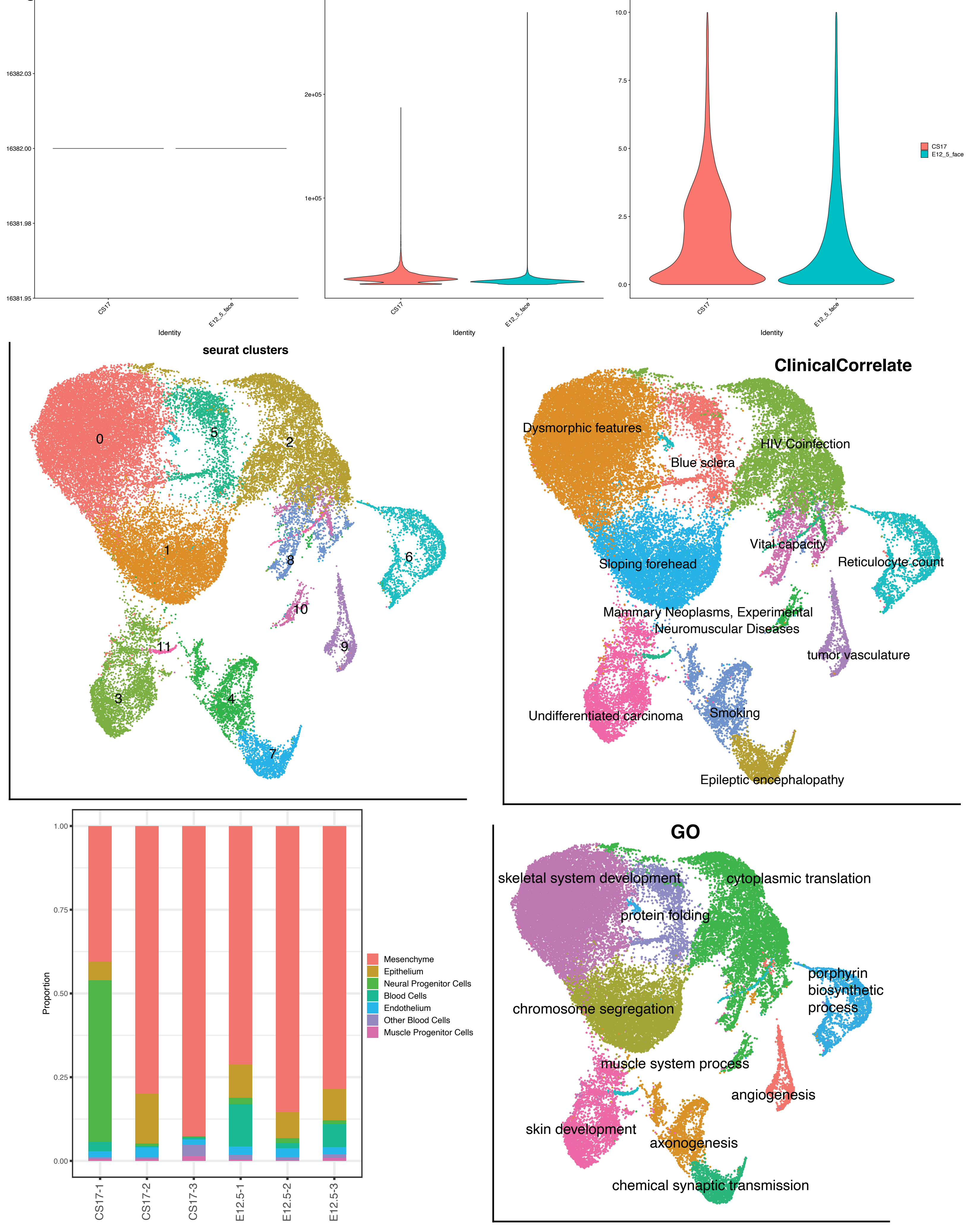

nFeature_RNA
nCount_RNA
percent.mt
16382.03
16382.00
16381.98
16381.95
2e+05
1e+05
10.0
7.5
5.0
2.5
0.0
CS17
E12_5_face
Identity
CS17
E12_5_face
seurat clusters
0
1
2
3
4
5
6
7
8
9
10
11
ClinicalCorrelate
Dysmorphic features
Blue sclera
HIV Coinfection
Vital capacity
Reticulocyte count
Sloping forehead
Mammary Neoplasms, Experimental
Neuromuscular Diseases
tumor vasculature
Undifferentiated carcinoma
Smoking
Epileptic encephalopathy
1.00
0.75
0.50
0.25
0.00
Proportion
CS17-1
CS17-2
CS17-3
E12.5-1
E12.5-2
E12.5-3
Mesenchyme
Epithelium
Neural Progenitor Cells
Blood Cells
Endothelium
Other Blood Cells
Muscle Progenitor Cells
GO
skeletal system development
cytoplasmic translation
protein folding
porphyrin
biosynthetic
process
chromosome segregation
muscle system process
angiogenesis
skin development
axonogenesis
chemical synaptic transmission

Figure S11. Quality Control Metrics of snRNA-seq of Human and Mouse Craniofacial Regions. Related to Figure 7. From Top left: Violin plots of total number of unique RNA species detected across indivdiual human (red) and mouse (blue) samples. Violin plots of total counts obtained in individual human and mouse samples. Violin plots of estimated percentage of mitochondrial reads from human and mouse samples. From Middle left: Original cluster ids as reported from Seurat for coprojected human and mouse snRNA-Seq data. Top Clinical Correlate category for each cluster based on marker genes for each cluster. From Bottom Left: Bar charts indicating proportion of each human or mouse replicate in each cluster identified by Seruat. Top Gene Ontology category for each cluster based on marker genes for each cluster.

Figure S12

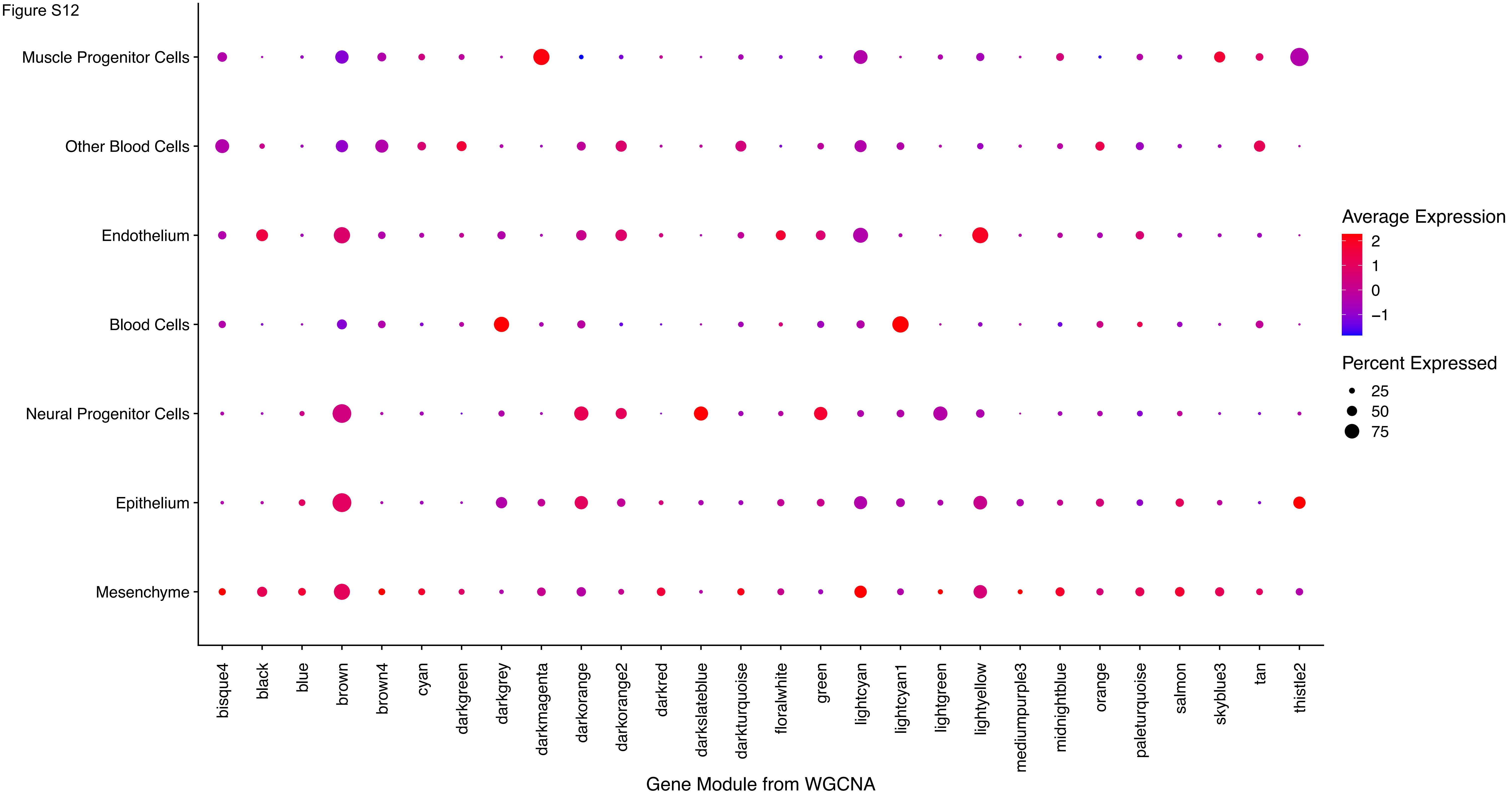
Muscle Progenitor Cells
Other Blood Cells
Endothelium
Blood Cells
Neural Progenitor Cells
Epithelium
Mesenchyme
bisque4
black
blue
brown
brown4
cyan
darkgreen
darkgrey
darkmagenta
darkorange
darkorange2
darkred
darkslateblue
darkturquoise
floralwhite
green
lightcyan
lightcyan1
lightgreen
lightyellow
mediumpurple3
midnightblue
orange
paleturquoise
salmon
skyblue3
tan
thistle2
Gene Module from WGCNA
Average Expression
2
1
0
−1
Percent Expressed
25
50
75

Figure S12. Module score dotplot of all WGCNA module hub genes across major snRNA-seq cell types. All remaining moudle scores per module across all cell types as from Figure 7D.
