# Supplementary material for: Integrative analysis of transcriptome dynamics during human craniofacial development identifies candidate disease genes
Source: Nat Commun. 2023 Aug 2;14:4623. doi: 10.1038/s41467-023-40363-1 (PMC10397224; doi:10.1038/s41467-023-40363-1)
Supplement: Supplementary file 3 — Description of Additional Supplementary Files [file 41467_2023_40363_MOESM3_ESM.pdf]

# Description of Additional Supplementary Files

**Supplementary Data 1: Embryo metadata and mouse tissue samples.** Tab 1: Metadata as reported by HDBR for embryonic sequencing samples generated by our lab (Columns 1 through 6). RIN scores determined in this study, batch numbers represent separate individuals generating the libraries. Chromatin state represents samples also used for chromatin state annotation by Wilderman et al 2018. Tab 2: Mouse tissue samples retrieved from recount3.

**Supplementary Data 2: Gini Analysis.** Tab 1: List of Gini scores and tissue specificity for each gene. Gini score (0 to 1) for 33073 genes across 34 tissues. Tab 2: Gene Ontology analysis of CF Gini Genes. Contains unadjusted (column labeled LogP) and adjusted p-value represented as a q-value (column labeled Log(q-value)). Tab 3: Disgenet analysis of CF Gini Genes. Contains unadjusted p-value (column labeled pvalue) and Benjamini-Hochberg adjusted p-values (column labeled FDR) calculated by the disgenet2r148 package in R. Tab 4: Disease Gene lists obtained from Disgenet and CleftGeneDB. Tab 5: TF Enrichment analysis of CF Gini Genes.

**Supplementary Data 3: Bed file of craniofacial specific enhancers.** Regions determined by chromatin state segmentation (ChromHMM) and include states 13, 14, 15 and 18. The second tab contains results from GREAT for these segments (McLean et al., 2010).

**Supplementary Data 4: Pairwise differential expression results.** Results from DESeq2 with raw scaled counts tables attached. Contains columns for both unadjusted (pvalue) and adjusted p-values (padj) as calculated by DESeq2. The last tab contains information for each gene and their cluster designation. Related to Figure 4.

**Supplementary Data 5: Gene ontology results of each WGCNA module.** Related to Figure 5A. Contains columns for both unadjusted (column labeled PValue) and several methods of multiple-test corrections were calculated by RDAVIDWebService155 (columns labeled Bonferroni, Benjamini & FDR).

**Supplementary Data 6: WGCNA results.** A list of genes assigned to each module separated by tabs.Integrative information included such as Gini status and known disease link. Related to Figure 5 and 6.

**Supplementary Data 7: Prioritized CF disease candidate list.** Tab 1: Prioritized genes along with a designation column that indicates if a gene is in the DisGeNET database for craniofacial-related diseases: Craniosynostosis, Cleft palate, Cleft lip, Craniofacial abnormalities. Tab 2: Genes from Bishop et al 2020 identified by our prioritization scheme and statistical enrichments.

**Supplementary Data 8: Single-Nuclei RNA-seq marker genes and gene ontology enrichment.** Results output from Seurat. Related to Figure 7.
