# Supplementary material for: Integrative analysis of transcriptome dynamics during human craniofacial development identifies candidate disease genes
Source: Nat Commun. 2023 Aug 2;14:4623. doi: 10.1038/s41467-023-40363-1 (PMC10397224; doi:10.1038/s41467-023-40363-1)
Supplement: Supplementary file 12 — Reporting Summary [file 41467_2023_40363_MOESM12_ESM.pdf]

nature portfolio

Corresponding author(s): Justin Cotney
Last updated by author(s): Jun 26, 2023

Reporting Summary

Nature Portfolio wishes to improve the reproducibility of the work that we publish. This form provides structure for consistency and transparency in reporting. For further information on Nature Portfolio policies, see our Editorial Policies and the Editorial Policy Checklist.

Statistics

For all statistical analyses, confirm that the following items are present in the figure legend, table legend, main text, or Methods section.

n/a	Confirmed	
☐	☒	The exact sample size (*n*) for each experimental group/condition, given as a discrete number and unit of measurement
☐	☒	A statement on whether measurements were taken from distinct samples or whether the same sample was measured repeatedly
☐	☒	The statistical test(s) used AND whether they are one- or two-sided<br>*Only common tests should be described solely by name; describe more complex techniques in the Methods section.*
☐	☒	A description of all covariates tested
☐	☒	A description of any assumptions or corrections, such as tests of normality and adjustment for multiple comparisons
☐	☒	A full description of the statistical parameters including central tendency (e.g. means) or other basic estimates (e.g. regression coefficient) AND variation (e.g. standard deviation) or associated estimates of uncertainty (e.g. confidence intervals)
☐	☒	For null hypothesis testing, the test statistic (e.g. *F*, *t*, *r*) with confidence intervals, effect sizes, degrees of freedom and *P* value noted<br>*Give P values as exact values whenever suitable.*
☒	☐	For Bayesian analysis, information on the choice of priors and Markov chain Monte Carlo settings
☒	☐	For hierarchical and complex designs, identification of the appropriate level for tests and full reporting of outcomes
☐	☒	Estimates of effect sizes (e.g. Cohen's *d*, Pearson's *r*), indicating how they were calculated

Our web collection on statistics for biologists contains articles on many of the points above.

Software and code

Policy information about availability of computer code

Data collection

Human Tissue Samples
Use of human embryonic tissue was reviewed and approved by the Human Subjects Protection Program at UConn Health (UCHC 710-2-13-14-03). Human craniofacial tissue was consented, collected, staged, and provided by the Joint Medical Research Council (MRC)/ Wellcome Trust Human Developmental Biology Resource (HDBR). Further documentation of all policies and ethical approvals for HDBR sample collection can be found at https://www.hdbr.org/ethical-approvals. Tissues were flash frozen upon collection and stored at −80°C. Upon thawing the samples were quickly inspected for intactness of the general craniofacial prominences. They were further dissected to exclude other tissues that might have been included initially such as eye spots or cardiac outflow tracts. The general regions that were collected are indicated in yellow in Figure 1A. All CS22 samples were processed by the laboratory of Axel Visel and retrieved from Facebase (Table S1).

Mouse Embryonic Tissue Samples
Use of mouse embryonic tissues was reviewed and approved by the UConn Health Institutional Animal Care and Use Committee (Protocol AP-2000061-0723). 8 week old wild type male and female C57BL6/J mice were obtained from Jackson Laboratory. Mice were housed according to recommendations by Jackson Laboratory with 12h light:dark cycle beginning at 7 a.m. The ambient temperature was maintained between 20 and 22°C and humidity was maintained at 40–60%. Mice were given ad libitum access to food and water. Timed matings were established by identification of vaginal plug the morning following housing of a single male with multiple female mice. Embryos were harvested from pregnant mothers at mid-day 12 days after identification of vaginal plug. Staging was confirmed by counting somites and comparing overall morphology to the Theiler Staging Criteria137. All embryos from a given litter were combined for individual biological replicates. Craniofacial prominences were collected in a very similar fashion to human samples and subsequently prepared for snRNA-Seq.

CNCC differentiation
Dissociated (using 40um filter) H9 ESCs were plated at 30,000 cells per cm2 in NCC media with 10 ul Rock Inhibitor and 10 uL of 3mM stock CHIR99021. NCC Media: 48.5 mL DMEM/F12 (Gibco#10565-018) 500uL 50U/mL penicillin, 500 uL 50U/mL streptomycin, 1mL B27. Media was changed daily, the rock inhibitor added on the day following plating and not after. Differentiation is complete by day 5.

RNA-seq
Frozen tissue samples were added to Qiazol (Qiagen) and subjected to mechanical disruption using a motorized pestle. Homogenates were then processed using the miRNeasy RNA extraction kit (Qiagen, 217004) with on-column DNAse treatment (Qiagen, 79254) according to the manufacturer's protocol. RNA integrity was checked using Agilent Tapestation 2200 with Agilent RNA analysis screentapes (Agilent Genomics, 5067-5576). RNA with RNA Integrity Number (RIN) scores preferably > 8.0 were used in the preparation of RNA-seq libraries. RNA-seq libraries were prepared from 100-200ng total RNA using the TruSeq stranded mRNA kit (Illumina, RS-122-2101) according to the manufacturer's instructions with the modification to use Superscript III Reverse Transcriptase enzyme (Invitrogen, 18080044) during the first strand cDNA synthesis step. Completed libraries were checked for quality and average fragment size using the Agilent Tapestation 2200 with D1000 screen tapes (Agilent Genomics, (5067- 5582). Molar concentration determined using NEBNext qPCR library quantification kit (NEB,E7630). Libraries were pooled and diluted to 1.8pm and sequenced on the NextSeq500 Illumina platform using 75bp paired end sequencing according to manufacturer's recommendations. Libraries were diluted to 4nM, pooled and denatured according to the instructions for Illumina NextSeq 550/500. Libraries were sequenced on the NextSeq 500 or 550 with settings for single-index, paired-end sequencing with 75 cycles per end.

snRNA-seq

Samples were mechanically disrupted into liquid suspensions, checked for viability and counted using Trypan blue staining. Nuclei were isolated and quantified following the established protocol (10x Genomics®). Samples were transferred to the Jackson Laboratories (Farmington, CT) Single Cell Biology Laboratory (SCBL) for processing which followed the Chromium Next GEM Single Cell Multiome ATAC + Gene Expression user guide from 10x Genomics®. Sequencing was done on an Illumina NovaSeq.

Data analysis

RNA-seq Data Processing

Human
Quality control was performed on RNA-seq reads using FastQC (v.0.11.7) and MultiQC (v.1.1)138. Trimming for adapters, quality and length was performed using Trimmomatic (v.0.36)139. Trimmed fastqs were aligned with Rail-RNA (v.0.2.4b)45 using human assembly GRCh38/hg38. RSeQC (v.4.0.0)140 was used to calculate the read distribution, gene body coverage and transcript integrity number (TIN) score141 (Figure 1B, S1A) . The coverage bigWig files output by Rail-RNA (v0.2.4b) were used as input for the generation of counts tables by following the instructions and pipeline from recount2 (https://github.com/leekgroup/recountcontributions), where the comprehensive Gencode v.25 annotation was used. The level 3 genes as defined by gencode were excluded. The recount rse_gene objects for each sample were combined into one rse_gene object and transformed with scale_counts from recount (v.1.8.2). The PCA plots in Figure 1B,S1, S2 were made using the prcomp function from the built-in R (v3.5.3) stats package on a DESeq2 (v.1.26.0)142 rlog transformation on the raw counts of the 18597 most highly expressed genes across all craniofacial samples generated in this study.

Mouse
Counts table for mouse data was collected and generated by the pipeline from Recount370 R package (v.1.8.0). 500 samples from 17 studies were gathered into a rse_gene object and scaled with transform_counts function of Recount370 (v.1.8.0). Gencode v.M23 annotation was used. Integrated counts table was further performed batch correction with RUVs in RUVSeq143 R package (v.1.3.2) A full listing of samples used in this analysis is provide in Table S1. After generating scaled and batch-corrected counts table, all pseudogenes and microRNA genes by Annotationdbi144 (v.1.60.0) were discarded, thereby 26379 genes were used for downstream analysis. For PCA plot in figure S2, we used pcaplot3d function from pcaExplorer145 package (v.2.24.0) and plotly function from plotly package (v. 4.10.1).

Human and Mouse Craniofacial data comparison
Trimmed fastqs of human craniofacial data (CS13~CS17) were aligned and annotated on GRCh38/Gencode v.26 with Monorail pipeline (v20220219) (https://github.com/langmead-lab/monorail-external) to build rse object in recount370. Mouse facial data were from ENCODE (GSE37909, GSE57230), collected by the recount370 database (compatible SRA ID: SRP013027, SRP013703). Each counts table was further performed scaling and batch correction, respectively. To combine human and mice data, only 1:1 orthologs between hg38 and mm10 were used. Thereby 15567 genes were used for every downstream analysis for human and mice craniofacial comparison, in log2-transformed values with offset 1.

snRNA-seq Data Processing

Human raw fastqs were aligned to Gencode37 using CellRanger (v6.1.2) and gene counts per cell were imported into Seurat146 (v3.2.0). Mouse raw fastqs were aligned to mm10 genome using CellRanger and gene counts per cell were imported into R. Each mouse mm10 counts table was converted to human Gencode37 orthologs using a 1:1 orthology table. Mouse and Human genes with no single ortholog were excluded from our analyses. Seurat146 (v.3.2.0) was used for filtering, merging of samples, scaling, normalization, dimensionality reduction (UMAP), and clustering. Clusters were functionally annotated using GO enrichment analysis in clusterProfiler147 (v.3.14.3) from marker genes of each cluster. Enrichment of the WGCNA module hub genes per cell type (Figure 7B,C) were calculated using Seurat146 function AddModuleScore (options bins=1, control=1000, seed = 1, search = TRUE). Coexpression UMAPs were calculated using FeaturePlot with blend =TRUE and order=TRUE. Detailed scripts of analyses can be found at our github (https://github.com/cotneylab/Embryonic_Gene_Expression_and_Chromatin_Dynamics/tree/master/Craniofacial_Transcriptomics).

For manuscripts utilizing custom algorithms or software that are central to the research but not yet described in published literature, software must be made available to editors and reviewers. We strongly encourage code deposition in a community repository (e.g. GitHub). See the Nature Portfolio guidelines for submitting code & software for further information.

Data

Policy information about availability of data

All manuscripts must include a data availability statement. This statement should provide the following information, where applicable:

- Accession codes, unique identifiers, or web links for publicly available datasets
- A description of any restrictions on data availability
- For clinical datasets or third party data, please ensure that the statement adheres to our policy

Data availability
The craniofacial bulk and single cell RNA-seq data generated in this study have been deposited in the Gene Expression Omnibus (GEO) database under accession code GSE197513 (https://www.ncbi.nlm.nih.gov/geo/query/acc.cgi?&acc=GSE197513) and Database of Genotypes and Phenotypes (dbGAP) under accession code phs002008 (https://www.ncbi.nlm.nih.gov/projects/gap/cgi-bin/study.cgi?study_id=phs002008.v1.p1). The raw data are available under restricted access for human genomics data privacy concerns, access can be obtained by application to dbGap.

Sequence and phenotype data for orofacial clefting whole genome sequencing is available from the (dbGaP) under study accessions phs002220.v1.p1, phs001168.v2.p2, phs001420.v1.p1, and phs000094.v1.p1 (https://www.ncbi.nlm.nih.gov/projects/gap/cgi-bin/study.cgi?study_id=phs002220.v1.p1,https://www.ncbi.nlm.nih.gov/projects/gap/cgi-bin/study.cgi?study_id=phs001168.v2.p2,
https://www.ncbi.nlm.nih.gov/projects/gap/cgi-bin/study.cgi?study_id=phs001420.v1.p1,https://www.ncbi.nlm.nih.gov/projects/gap/cgi-bin/study.cgi?study_id=phs000094.v1.p1
).
The single nuclei and bulk RNA-Seq data can be interactively explored at http://cotneyweb.cam.uchc.edu/craniofacial_cs17_e12_5/ and http://cotneyweb.cam.uchc.edu/craniofacial_bulkrna/ respectively.

Mouse craniofacial gene expression generated by ENCODE were retrieved from GEO through accessions GSE37909 and GSE57230.
A full listing of all data utilized from all 500 mouse samples retrieved from Recount 3 is available in Table S1.

Human CNCC RNA-Seq data were obtained from GEO accession GSE70751, SRX numbers SRR2096446 through SRR2096451.

Research involving human participants, their data, or biological material

Policy information about studies with human participants or human data. See also policy information about sex, gender (identity/presentation), and sexual orientation and race, ethnicity and racism.

Reporting on sex and gender	This research related to gene expression does not include human subjects as determined by UCHC IRB, but we do provide sex of each embryo in Table S1.<br><br>Whole genome sequencing was performed at the Center for Inherited Disease Research at Johns Hopkins University (Baltimore, MD) and the Broad Institute as described previously126,127.<br><br>126. Bishop, M. R. et al. Genome-wide Enrichment of De Novo Coding Mutations in Orofacial Cleft Trios. American journal of human genetics 107, 124-136 (2020).<br>127. Robinson, K. et al. Trio-based GWAS identifies novel associations and subtype-specific risk factors for cleft palate. medRxiv, 2023.03.01.23286642 (2023).
Reporting on race, ethnicity, or other socially relevant groupings	Whole genome sequencing was performed at the Center for Inherited Disease Research at Johns Hopkins University (Baltimore, MD) and the Broad Institute as described previously126,127.<br><br>126. Bishop, M. R. et al. Genome-wide Enrichment of De Novo Coding Mutations in Orofacial Cleft Trios. American journal of human genetics 107, 124-136 (2020).<br>127. Robinson, K. et al. Trio-based GWAS identifies novel associations and subtype-specific risk factors for cleft palate. medRxiv, 2023.03.01.23286642 (2023).
Population characteristics	Whole genome sequencing was performed at the Center for Inherited Disease Research at Johns Hopkins University (Baltimore, MD) and the Broad Institute as described previously126,127.<br><br>126. Bishop, M. R. et al. Genome-wide Enrichment of De Novo Coding Mutations in Orofacial Cleft Trios. American journal of human genetics 107, 124-136 (2020).<br>127. Robinson, K. et al. Trio-based GWAS identifies novel associations and subtype-specific risk factors for cleft palate. medRxiv, 2023.03.01.23286642 (2023).
Recruitment	Whole genome sequencing was performed at the Center for Inherited Disease Research at Johns Hopkins University (Baltimore, MD) and the Broad Institute as described previously126,127.<br>126. Bishop, M. R. et al. Genome-wide Enrichment of De Novo Coding Mutations in Orofacial Cleft Trios. American journal of human genetics 107, 124-136 (2020).

127. Robinson, K. et al. Trio-based GWAS identifies novel associations and subtype-specific risk factors for cleft palate. medRxiv, 2023.03.01.23286642 (2023).

Ethics oversight

Use of human embryonic tissue for gene expression profiling was reviewed and approved by the Human Subjects Protection Program at UConn Health (UCHC 710-2-13-14-03).

Whole genome sequencing of orofacial clefting trios was approved by the institutional review board of Emory University (protocol IRB00098814).

EBF3 related work was approved by the institutional review board of Baylor College of Medicine (BCM protocol H-47546) and was conducted in accordance with the ethical standards of this institution's committee on human research and international standards.

Note that full information on the approval of the study protocol must also be provided in the manuscript.

Field-specific reporting

Please select the one below that is the best fit for your research. If you are not sure, read the appropriate sections before making your selection.

[x] Life sciences [ ] Behavioural & social sciences [ ] Ecological, evolutionary & environmental sciences

For a reference copy of the document with all sections, see nature.com/documents/nr-reporting-summary-flat.pdf

Life sciences study design

All studies must disclose on these points even when the disclosure is negative.

Sample size

Number of human embryonic tissues samples was largely determined by availability. We limited our analysis to timepoints where we could acquire at least 3 biological replicates. All sample info related to sex, karyotype, etc are detailed in Table S1.

Data exclusions

No human data was excluded from our analysis.

Replication

Our findings were attempted to be replicated by comparisons with mouse gene expression data and previous human ChIP-Seq data. We found good agreement in craniofacial-biased gene expression, overall patterns of gene expression across craniofacial development, and strong positive correlations with number of craniofacial specific enhancers that target a gene.

Randomization

This is not relevant to our study as we were characterizing normal human development. Samples were distributed across stages based on assigned Carnegie state by the Human Developmental Biology Resource.

Blinding

Blinding is not relevant to our study as we did not have experimental groups.

Reporting for specific materials, systems and methods

We require information from authors about some types of materials, experimental systems and methods used in many studies. Here, indicate whether each material, system or method listed is relevant to your study. If you are not sure if a list item applies to your research, read the appropriate section before selecting a response.

Materials & experimental systems

n/a	Involved in the study	
[x]	[ ]	Antibodies
[ ]	[x]	Eukaryotic cell lines
[x]	[ ]	Palaeontology and archaeology
[ ]	[x]	Animals and other organisms
[x]	[ ]	Clinical data
[x]	[ ]	Dual use research of concern
[x]	[ ]	Plants

Methods

n/a	Involved in the study	
[x]	[ ]	ChIP-seq
[x]	[ ]	Flow cytometry
[x]	[ ]	MRI-based neuroimaging

Eukaryotic cell lines

Policy information about cell lines and Sex and Gender in Research

Cell line source(s)	WiCell H9
Authentication	Confirmed normal haplotype by chromosomal microarray
Mycoplasma contamination	Cell lines tested routinely for mycoplasma contamination using commercially available kits from ThermoScientific.
Commonly misidentified lines (See ICLAC register)	NA

Animals and other research organisms

Policy information about studies involving animals; ARRIVE guidelines recommended for reporting animal research, and Sex and Gender in Research

Laboratory animals	8 week old wild type male and female C57BL6/J mice were obtained from Jackson Laboratory. Mice were housed according to recommendations by Jackson Laboratory with 12h light:dark cycle beginning at 7 a.m. The ambient temperature was maintained between 20 and 22°C and humidity was maintained at 40–60%. Mice were given ad libitum access to food and water. Timed matings were established by identification of vaginal plug the morning following housing of a single male with multiple female mice. Embryos were harvested from pregnant mothers at mid-day 12 days after identification of vaginal plug. Staging was confirmed by counting somites and comparing overall morphology to the Theiler Staging Criteria137. All embryos from a given litter were combined for individual biological replicates. Craniofacial prominences were collected in a very similar fashion to human samples and subsequently prepared for snRNA-Seq.
Wild animals	NA
Reporting on sex	Multiple embryos from the same litter were included for each snRNA-Seq replicate. We confirmed presence of both male and female cells in each replicate through Xist gene expression.
Field-collected samples	NA
Ethics oversight	All animal work was reviewed and approved by the UConn Health Institutional Animal Care and Use Committee. Protocol AP-2000061-0723.

Note that full information on the approval of the study protocol must also be provided in the manuscript.
